# Alcohol consumption among partners of pregnant women in Sweden: a cross sectional study

**DOI:** 10.1186/s12889-016-3338-9

**Published:** 2016-08-02

**Authors:** Hjördis Högberg, Janna Skagerström, Fredrik Spak, Per Nilsen, Margareta Larsson

**Affiliations:** 1 Department of Women’s and Children’s Health, Uppsala University, Uppsala, Sweden; 2 Department of Health and Medical Science, Division of Community Medicine, Linköping University, Linköping, Sweden; 3 Social medicine, University of Gothenburg, Göteborg, Sweden; 4 Psykiatri Skåne, Divisionsledningen, Baravägen 1, S-22185 Lund, Sweden

**Keywords:** Alcohol consumption, AUDIT-C, Drinking context, Generations, Health promotion, Life cycle perspective, Partner, Pregnancy, Social support

## Abstract

**Background:**

Antenatal care in Sweden involves a visit in pregnancy week 6–7 for counseling about lifestyle issues, including alcohol. The aim of this study was to investigate alcohol consumption among partners of pregnant women, their motives for changing drinking patterns when becoming a parent and their perceptions of the midwife’s counseling about alcohol.

**Method:**

The study was conducted at 30 antenatal care centers across Sweden in 2009–2010. All partners who accompanied a pregnant women in pregnancy week >17 were asked to participate. The questionnaire included questions on alcohol consumption.

**Results:**

Questionnaires from 444 partners were analyzed. Most, 95 %, of the partners reported alcohol consumption before pregnancy; 18 % were binge drinking (6 standard drinks or more per occasion, each drink containing 12 grams of pure alcohol) at least once every month during the last year. More than half, 58 %, of all partners had decreased their alcohol consumption following pregnancy recognition and a higher proportion of binge drinkers decreased their consumption compared to non-frequent binge drinkers (*p* = 0.025). Their motives varied; the pregnancy itself, fewer social gatherings (potentially involving alcohol consumption) and a sense of responsibility for the pregnant partner were reported. Of the partners, 37 % reported support for decreased drinking from others (pregnant partner, parents, friend or workmates). Further, most partners appreciated the midwife’s counseling on alcohol.

**Conclusion:**

A majority of partners decreased their alcohol consumption in transition to parenthood, which also appears to be a crucial time for changing alcohol-drinking patterns. The partners with higher AUDIT-C scores reported more support for decreased drinking. Most partners appreciated the midwife’s talk about alcohol and pregnancy and those who filled out AUDIT in early pregnancy reported that the counseling was more engaging. During pregnancy it is possible to detect partners with high alcohol consumption, and promote interventions for decreased drinking, also for the partners. Written information addressing alcohol use and directed to partners is needed.

## Background

Alcohol affects the overall health and is, therefore, important from a public health perspective globally. After tobacco and high blood pressure, alcohol is the third largest risk factor for men (and seventh largest risk factor for women) for mortality and premature death in developed countries [1]. Pre pregnancy knowledge about alcohol and pregnancy is important to enable changes in alcohol consumption in order to avoid harmful effects on the fetus [2, 3]. Similar to many other countries, Swedish maternity care recommends that all pregnant women should abstain from alcohol during pregnancy [4–7]. However, one review concludes that: “Dose–response relationship indicates that heavy alcohol consumption during pregnancy increases the risks whereas light to moderate alcohol consumption shows no effect [8]. The evidence on whether there are safe levels of alcohol consumption during a pregnancy remains inconclusive.

Research focusing on the partner’s alcohol use before and during pregnancy is scarce in comparison with studies about pregnant women [9–13]. Some studies have shown that younger expecting fathers reduce their alcohol consumption more than older fathers [14, 15]. More recent research has shown that first-time fathers reduced their alcohol consumption more than fathers who already had children [16]. One recently published longitudinal study found that the quantity of alcohol drinking of the expected child’s father was a predictive variable for maternal risk drinking during pregnancy [17]. Another study reported a nine times higher risk for Fetal Alcohol Spectrum Disorder (FASD) in first year school–children if there was an alcohol problem in the family [18].

The relevance of social support for behavior change is reflected in numerous social-cognitive theories. For example, the Self-Determination Theory (SDT) [19] points to the importance of social relations to facilitate more intrinsically motivated behaviors which are performed with more persistence and better quality than behaviors guided by extrinsic factors [20]. Better results have been achieved when treatment focuses on support for the clients’ own decisions and autonomy [20]. Midwives in Sweden are generally trained in Motivational Interviewing (MI), and many use this method in the promotion of an alcohol-free pregnancy. MI in combination with SDT may be used to create a theory-based dialogue with parents-to-be about an alcohol free pregnancy and responsible drinking during parenthood [21, 22]. Similarly, Protection Motivation Theory (PMT) recognizes the importance of self-efficacy, i.e. the extent of one’s belief in one’s own ability to reach goals, which is influenced by social persuasion, either as direct encouragement or discouragement from another person [23, 24]. PMT has been applied in many health campaigns to prevent FASD [25]. Social support for decreased drinking may be important both for the partner and the pregnant woman [26–28]. Although previous studies have yielded important information about the correlates of prenatal alcohol use, questions regarding a partner’s influence remain [28].

Parents-to-be generally wish the partner to be more included in counseling and care and that the health care provider should focus on the whole family [29, 30]. Different studies highlight the need for further investigation and interventions with both parents-to-be about alcohol, pregnancy, and parenthood [16, 31–35].

The objective of this study was thus to investigate alcohol consumption among partners who attend ANC together with the pregnant women, if they have changed their alcohol consumption during their partner’s pregnancy and if so, the reasons for this change. Further aims were to investigate the partner’s support from others for decreased drinking and their perception of the midwife’s counseling about alcohol.

## Methods

### Study setting and sample

The present study a part of a research study on alcohol consumption of both pregnant women and their partners. Findings concerning the women are presented elsewhere [36]. In Sweden almost all pregnant women visit a midwife seven to eight times according to a basic program. The partner is always welcome to join the pregnant woman, and many partners do so at least at some of the visits. At the first visit the midwife has a dialogue with the pregnant woman concerning different lifestyle issues including alcohol use and in most regions in Sweden the woman is asked to fill out AUDIT. Written information is provided, but the brochure does not address the partner’s alcohol use.

The study was conducted at 30 ANCs in Sweden from November 2009 to December 2010. Each clinic collected data during a 4-week period. A strategic selection of ANCs across Sweden was recruited based on the distribution of pregnant women in 2008. The goal was to recruit a representative sample of clinics based on two dimensions: geographic location and population size (major city, >200 000 inhabitants; medium-sized city, 50 000–200 000 inhabitants; or other city, <50 000 inhabitants or rural area). All pregnant women with a scheduled consultation in pregnancy week 18–42 were asked to complete a questionnaire. If the woman’s partner accompanied her to the consultation he/she was asked to participate in the present study. In total 1693 pregnant women were informed about the study and 1637 accepted to participate. Further information about the setting can be found in the paper about the pregnant women [36]. The female non-responders were younger than the participants (*p* < 0.001) but did not differ regarding parity, pregnancy week, or number of visits in antenatal care [36].

### Data collection

The available partners were asked to fill in the questionnaire in the waiting room (where no midwife was present). When completed, the questionnaire envelope was sealed and put into a box, thus assuring blindness to the midwife. In total, 445 partners participated, but one questionnaire was excluded due to inconsistent responses. In total 444 questionnaires were analyzed. No information is available about the number of partners who were not invited or about the number of partners who were invited, but declined participation.

### Questionnaire and study variables

The partner questionnaire aimed to measure and explore the partners’ alcohol consumption, possible changes in drinking pattern, reasons for decreased drinking, social support for alcohol free pregnancy and experiences and perceptions about the midwives’ counseling.

The questionnaire was in Swedish and consisted of 12 questions; socio demographics, alcohol consumption during last year measured with AUDIT-C [37] (see Table 1), if they had changed alcohol use during the partners pregnancy and if so, what motives they presented for this change in an open-ended question. Further, the information included if they had received social support from others (partner, father, mother, friend, workmate) for changing alcohol consumption during pregnancy, if they had been asked to fill out any AUDIT [38] at ANC, and if they had participated when their pregnant partner was asked to fill out AUDIT. Seven statements investigated the opinions about the counseling on alcohol and pregnancy provided by the midwife with four Likert-scale response alternatives ranging from “totally agree” to “disagree”, also offering a fifth response alternative; “no opinion”. The last question was about partner involvement: “Did you want to be more involved in the counseling about alcohol?”(yes/no).

**Table 1:**
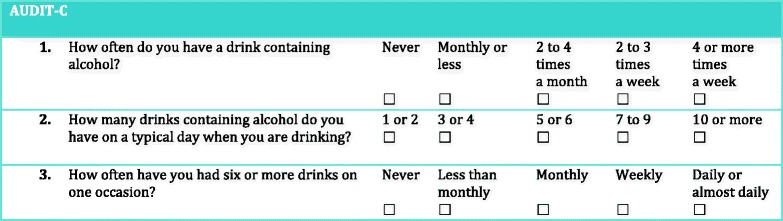
AUDIT-C questions

Alcohol consumption over the last year was measured using AUDIT-C. The AUDIT-C is scored on a scale from 0–12. Each AUDIT-C question has 5 response alternatives (Table 1). Points allotted are: 0 points, 1 point, 2 points, 3 points, 4 points. In men, a score of 5 or more is considered as indicatiative of hazardous drinking and at higher values also of alcohol use disorder, and the corresponding score in women is 4 or more [37, 39]. Generally, the higher the score, the more likely it is likely that the person’s drinking is affecting his or her health and safety. Partners who answered that they had not been drinking in the past 12 months were defined as abstainers.

### Data analysis

Data were entered and analyzed in SPSS 22. Before analysis some variables were recoded and/or collapsed into fewer response alternatives. Age was coded into three age groups; ≤ 24, 25–34 and ≥ 35. Education was collapsed into three alternatives: elementary school, high school, and university. Occupation was collapsed into two categories: occupied (including studying and parental leave) or not occupied (unemployed, sick leave). Number of children was coded as first-time parent or already having children. Four alternatives of alcohol consumption were constructed: abstainers, not binge drinking, binge drinking and frequent binge drinking.

The Mann–Whitney test and the Kruskal-Wallis test were used to investigate differences in AUDIT-C sum with respect to other variables such as: age, education, region, occupation, first time parent vs. partners with previous children, city size, completed AUDIT at ANC, quantity and frequency of drinking last year, frequency of binge drinking and frequent binge drinking last year, social support for decreased drinking, change of alcohol use during pregnancy, and if the partner wanted to be more involved in counseling about alcohol. The significance level was chosen as *p* < 0.05.

The seven items about the midwife’s counseling is not a validated instrument, so we did not collapse the items into a sum score and have only presented results from each individual item. However, we did examine the internal consistency, with Cronbach’s Alpha being 0.95, which means that there was a high internal consistency among these items.

The responses to the open-ended question about motives for decreased drinking were analyzed by summative content analysis [40]. All statements were read and then two of the authors independently sorted the responses into categories. Some comments were split and sorted into two separate categories. These categories were then discussed and closely examined by three of the authors and some minor adjustments in the labeling of the categories took place. The final six categories are presented in the result section with some illuminating examples.

### Ethics

At the end of the consultation the midwife gave an anonymous questionnaire to the woman and another anonymous questionnaire to the partner together with written and verbal information about the purpose of the study. Each participant as informed that participation in the study was voluntary and would not affect the future care in any way. The participants provided their consent by filling out and handing in the questionnaire.

The study was approved by the Regional Ethical Board in Linköping (Dnr M178-09).

## Results

### Sample characteristics

All partners came to the ANC together with the pregnant woman at one scheduled visit after pregnancy week 18. Table 2 presents the characteristics of the participants in relation to their AUDIT-C sum.

**Table 2:** Characteristics of the participants in relation to their AUDIT-C sum

Alcohol intake the last year	Partners *n* (%)	Mean AUDIT-C sum	*p*-value
Age			*p* = 0.292**
20–24	33 (7.8)	4.03	
25–34	261 (61.5)	3.76	
35-	130 (30.7)	3.51	
Education			*p* = 0.000**
Elementary school	21 (4.9)	4.29	
High school	231 (54.5)	3.93	
University	172 (40.6)	3.33	
Occupation			*p* = 0.860*
Employed	403 (95.0)	3.70	
Not employed	21 (5.0)	3.67	
Children			*p* = 0.250*
First time parent	303 (72.3)	3.77	
Has previous children	116 (27.7)	3.59	
City size			*p* = 0.842*
> 200.000	122 (28.8)	3.73	
< 200.000	302 (71.2)	3.69	
Region			*p* = 0.034**
North Sweden	180 (42.5)	3.79	
Mid Sweden	103 (24.3)	3.97	
South Sweden	141 (33.3)	3.40	

*Mann–Whitney *U* Test, **Kruskal-Wallis Test

### Alcohol use during last year

Most partners (95.0 %) used alcohol during the last year. Partners’ AUDIT-C results are shown in Table 3. More than half, 58 %, had decreased their alcohol consumption since pregnancy recognition and a higher proportion of frequent binge drinkers (who binge-drank every month or more often) decreased their alcohol consumption compared to non-frequent binge drinkers (*p* = 0.025). The AUDIT-C mean score was 3.70 (range 0.00–11.00) and the median score was 4.0. Partner with elementary and high school education reported higher AUDIT-C mean than partners with university education. About a fourth of the partners (27.4 %) had a score ≥5.0, indicating risk for hazardous drinking. The group of partners who reported support from others for changing alcohol consumption had a slightly higher AUDIT-C mean score 3.95 compared to 3.58 among those who did not report any support from others (*p* = 0.028). Partners’ AUDIT –C sum in relation to other variables is shown in Table 4.

**Table 3:** Results for partners AUDIT-C

AUDIT-C:	
1. How often do you have a drink containing alcohol? * n* = 443	*n* (%)
Never	22 (5.0)
Monthly or less	157 (35.4)
2–4 times a month	225 (50.7)
2–3 times a week	35 (7.9)
Four or more times a week	4 (0.9)
2. How many drinks containing alcohol do you have on a typical day when you are drinking? * n* = 425	*n* (%)
1–2 glasses	170 (40.0)
3–4 glasses	132 (31.1)
5–6 glasses	88 (20.7)
7–9 glasses	26 (6.1)
10 glasses or more	9 (2.1)
3. How often have you had six or more drinks on one occasion? * n* = 440	*n* (%)
Never	105 (23.9)
More seldom than once a month	254 (57.7)
Once a month, or 2–3 times a month	72 (16.4)
Each week	8 (1.8)
Daily or almost daily	1 (0.2)

**Table 4:** Partners’ AUDIT –C sum in relation to other variables

	Partners *n* (%)	Mean AUDIT-C sum	*p*-value
Completed AUDIT at ANC:			*p* = 0.355*
Yes	208 (50.5)	3.65	
No	204 (49.5)	3.77	
Drinking last year:			*p* = 0.014**
Decreased drinking	239 (57.9)	3.94	
The same drinking	171 (41.4)	3.48	
Increased drinking	3 (0.7)	6.00	
Social support for decreased drinking:			*p* = 0.011*
Yes	163 (40.3)	3.95	
No	241 (59.7)	3.57	
Partner wanted to be more involved in counseling about alcohol:			*p* = 0.225*
Yes	4 (1.0)	4.75	
No	386 (99.0)	3.70	

*Mann–Whitney *U* Test, **Kruskal-Wallis Test

### Binge drinking

Three out of four partners, 76 %, reported some binge drinking (6 standard glasses/occasion, each drink containing 12 grams of pure alcohol) and 28.9 % reported binge drinking (5–6 standard glasses) on a typical day when drinking alcohol. Almost one out of five partners (18.4 %) reported binge drinking at least once a month and 2.0 % reported weekly binge drinking. Binge drinking is shown in Table 3. We found no difference in binge drinking between first time parents and those who already had children.

### Alcohol use during pregnancy

More than every second partner (58 %) decreased drinking during pregnancy. Out of the partners (*n* = 240) who reported decreased drinking during last year, 22.9 % (*n* = 55) still reported frequent binge drinking (every month or more often). A higher proportion of frequent binge drinkers decreased their alcohol consumption compared to non-frequent binge drinkers (*p* = 0.025).

### Motives for decreased drinking

In connection with the partner’s pregnancy, 58 % (*n* = 240) of the partners decreased drinking. Most of them filled out an open-ended question about the motives for their decreased drinking. These motives were sorted into different categories presented in Table 5.

**Table 5:** Partners’ motives for decreased drinking

Motives for decreased drinking:	*N* = 201* (%)
Pregnancy, support and solidarity with “my” pregnant partner.	99 (49.3)
Alcohol should be consumed in fellowship.	69 (34.3)
More responsibility.	69 (34.3)
Fewer opportunities to drink alcohol at parties.	24 (11.9)
Does not feel right to be drinking now.	23 (11.4)
Coming child/parenthood.	8 (4.0)

*N = 201 participants motivated their decreased drinking and 92 of those gave more than one motive

*Pregnancy, support, and solidarity with “my” pregnant partner:* were the most commonly given motives for decreased drinking. This category also included comments referring to solidarity and support, also expressions of sympathy belonged here:

“Because my wife can’t drink so it’s not fair or appropriate for me to drink without her.”

*Alcohol should be consumed in fellowship (with partner and friends):* In this category the partner referred to the use of alcohol as a social activity with partner and friends, and if the pregnant woman abstained from alcohol there was no opportunity for the partner to drink alone:

“Alcohol should be enjoyed in company”.

*More responsibility (for my own health, matured, to drive the car, to be sober, alert):* Comments about the partner’s own personal development, maturity, health, to be sober and alert in case something would happen were sorted in this category: “I take the opportunity to be extra healthy.”

*Fewer opportunities to drink alcohol at parties (with friends or others, to get drunk):* In this category we sorted comments about the decrease in situations where alcohol is consumed:

“When we do not go to parties together, it (the decrease, authors note) will automatically happen”.

*Does not feel right to be drinking now (explicitly expressed different feelings):* All comments in this category involved feelings referring to the partner himself (or herself), expressed as positive without alcohol, feeling natural or avoiding negative emotions by abstaining or reducing alcohol use:

“It feels right (to abstain from alcohol)”.

*Coming child/parenthood (explicitly mentioned child, parenthood, or my family):* All comments in this category related to the coming child/parenthood:

“One has a responsibility for the kids. There is no need for alcohol when being with them”.

### Support from others for decreased drinking

More than every third partner (37.4 %, *n* = 166) reported support from others (e.g. pregnant partner, parents, friend or workmates) for decreased drinking during pregnancy, 55.5 % (*n* = 244) reported no support for decreased drinking and 6.2 % did not answer this question. There was no difference in binge drinking in relation to whether the partners reported support from others or not (*p* = 0.068).

### AUDIT

Half (50.1 %) of the partners reported that they had filled out AUDIT during an ANC visit in early pregnancy, 28.0 % of them (*n* = 122) reported decreased drinking and 19.1 % (*n* = 83) did not change drinking, whereas 0.5 % (*n* = 2) reported increased drinking. Similar proportions were found among the partners, who had not filled out AUDIT (26.2, 19.5 and 0.2 % respectively). Of all the partners, 39.1 % (*n* = 170) reported that they had not changed drinking, and 14.5 % (*n* = 25) of them reported frequent binge drinking.

Partners who reported that AUDIT was filled out together with the pregnant woman in early pregnancy reported to a higher extent that the counseling from the midwife had been engaging (*n* = 156, 77.2 %) compared to those partners who had not filled out AUDIT with the partner (*n* = 107, 61. 8 %), *p* = 0.008.

### Partners’ perception of the midwife’s counseling about alcohol during pregnancy

Most partners (90.4 %) did not want to be more involved in the discussion about alcohol, but they generally appreciated the midwife’s counseling about alcohol and pregnancy. Several participants did not answer the questions, probably because they had not participated in any alcohol counseling at ANC. See Table 6.

**Table 6:** Partner’s perception about the midwife’s counseling about alcohol

*N* = 444	Totally agree,	Largely agree	Agree to some extent	Disagree	No opinion	Missing
*n* = (%)	*n* = (%)	*n* = (%)	*n* = (%)	*n* = (%)	*n* = (%)	*n* = (%)
I remember the conversation.	165 (37.1)	97 (21.8)	44 (9.9)	7 (1.6)	71 (16.0)	60 (13.5)
*n* = 384 (86.5)						
I have got new knowledge.	29 (6.5)	40 (9.0)	100 (22.5)	126 (28.4)	88 (19.8)	61 (13.7)
*n* = 383 (86.3)						
The midwife talked in a good way.	203 (45.7)	92 (20.7)	20 (4.5)	2 (0.5)	68 (15.3)	59 (13.3)
*n* = 385 (86.7)						
The conversation/s has been engaging.	89 (20.0)	103 (23.2)	77 (17.3)	23 (5.2)	91 (20.5)	61 (13.7)
*n* = 383 (86.3)						
The conversation/s has not been intrusive.	253 (57.0)	18 (4.1)	14 (3.2)	5 (1.1)	92 (20.7)	62 (14.0)
*n* = 382 (86.0)						
The midwife was professional/well skilled.	183 (41.2)	100 (22.5)	29 (6.5)	2 (0.5)	71 (16.0)	59 (13.3)
*n* = 385 (86.7)						
The conversation/s about alcohol was not too long.	237 (53.4)	36 (8.1)	13 (2.9)	5 (1.1)	94 (21.2)	59 (13.3)
*n* = 385 (86.7)						

Cronbach’s Alpha for the seven items: 0.95

## Discussion

This paper shows that partners of pregnant women appreciated midwife-initiated counseling about alcohol. Many of the partners had decreased alcohol use in relation to the partner’s pregnancy and their motives varied; solidarity with the pregnant women, fewer occasions for social drinking and concern for their own health were most prominent. This is in line with research regarding smoking cessation in becoming fathers [41, 42].

More than half of the partners reported decreased drinking during pregnancy and the same pattern is reported in a study from Norway [16]. Other studies have shown that younger partners and first time parents are more willing to reduce drinking during pregnancy [14], but this was not confirmed in our study. Possible explanations may be that even first time partners nowadays tend to be older, or that partners accompanying a pregnant woman to an ANC are more engaged in the pregnancy and have other drinking habits than partners who do not visit the ANC. Partners who reported decreased alcohol use commented that the pregnancy was the most important motive for the change, together with less opportunities for social drinking and responsibility for own health. A previous study showed that becoming fathers appreciated to receive support for their own smoking cessation, and not only through the pregnant partner [42]. The same pattern may exist in relation to alcohol, because the partners in this study were more pleased with the counseling if they had filled out an AUDIT themselves.

The participants in this study generally reported lower AUDIT-C sums than was found in another Swedish study with data collected in 2003–2004 which showed AUDIT-C mean score 7.72 if the partner reported alcoholism in the family, and 6.68 if not (*p* < 0.01) [43]. This discrepancy may depend on a general time trend of decreased drinking during pregnancy or that the samples in the studies were different. In this study partners with lower education reported higher AUDIT-C mean sum and were also binge drinking to a higher extent. This group difference might be taken into account during the dialogue about alcohol. Still, the mean AUDIT-C scores for all education groups of partners were lower than the cut-off score for hazardous drinking in men.

More than one of three partners of the pregnant women reported support from others for decreased alcohol drinking during pregnancy. Most pregnant women in the women’s study [36] claimed that they received support from others, as did also the pregnant women in another previous study [43]. The group of partners who reported support from others for changing alcohol consumption had a higher AUDIT-C score compared those who did not report any support from others. From a public health perspective it is good that those who drink more also receive more support for a decrease. Other authors argue that the effectiveness of public health interventions with the aim to increase awareness about and reduce alcohol consumption among pregnant women cannot be assessed because of the paucity of studies and highlight the need of public health intervention [44].

The support for decreased drinking reported by the partners in this study is in line with social support theories, for example The Protection Motivation Theory (PMT) [25] and The Self Determination Theory (SDT) [19]. The partner is in most cases the most significant other for a pregnant woman, so the way he/she discusses and acts in relation to alcohol is most likely influential also for the pregnant woman’s own behavior change. A previous study showed that social support for changing drinking habits is important for some couples [43]. No studies were found about social support theories applied in relation to alcohol during pregnancy or with interventions directed to both parents-to-be, but several studies claim it is important for both parties in the couple to receive support from partner and others for decreased drinking, if needed [26–28]. Partners in this study reported their own health, increased maturity and a new responsibility as motives for decreased alcohol consumption. We interpret such motives as signs of increased self-efficacy and self-determination. Few studies have explicitly linked intervention strategies and theories of behavioral change in primary care settings [45]. Different theories might be combined when developing a theoretical framework for midwives’ dialogue about alcohol in a life cycle perspective with both parents-to-be [21, 22].

Partners generally appreciated the midwife’s counseling, since the majority agreed to the statements about the counseling, and remembered most, or the whole, of the conversation. Surprisingly, the partners reported that they had only received little new knowledge. This may indicate that they already had good knowledge, acquired from different arenas, or that knowledge-transfer was not the most vital part of the encounter. Many partners appreciated the manner in which the midwives conveyed the counseling, and those who filled out AUDIT in early pregnancy appreciated the midwives conversation to a higher extent. Midwives in Sweden are mostly well educated for health promotion in early pregnancy including how to use MI [22, 46] and this may have facilitated the alcohol counseling. Most partners agreed that the midwives were professional and this can be seen as a trust in the midwife’s competency. Most partners also considered that the midwives used appropriate time for the counseling, which indicates that midwives in general have a good understanding of the couple’s need and used the time during the visit well.

Having a dialogue about alcohol, pregnancy and parenthood with all parents-to be during all pregnancies may be a preventive measure because it may detect ongoing or earlier alcohol problems in the family. This alcohol dialogue between the couple and the midwife can take its starting point from the couple’s own alcohol experience. It is also possible to reflect about alcohol and social norms and habits and how to cope with changing life circumstances during pregnancy and parenthood [44, 47]. An earlier study showed that more than one of ten pregnant women had ever been worried about own and partner’s drinking and also that the partners reported worries about their own drinking to the same extent. Furthermore four out of five couples affirmed that support for an alcohol-free pregnancy and decreased alcohol consumption (also for the partner) had prepared the couple for joint parenthood [43].

Further studies are needed to evaluate how health promotion and primary prevention in relation to alcohol and pregnancy could be communicated and introduced already pre-pregnancy to both women and men separate or together, with parents-to-be, and among health professionals.

### Strengths and weaknesses

The strength of this study is the widespread geographic distribution of the participants, because earlier research has found that drinking among pregnant women in big cities is different compared with urban areas and country areas. A weakness is that only the partners who accompanied the woman to an ANC visit participated in the study and the total sample corresponds to 27 % of the pregnant women. It is possible that partners who did not come to ANC with their partners differed from the study sample. If this was the case, alcohol use could be even higher than reported in this paper. The results of this study may, therefore, only be generalized to partners of pregnant women participating in any visit at ANC.

Another weakness is that no questions about alcoholism/alcohol dependence/addiction in the family were included. The questionnaire did not contain any question about smoking so no comparison between alcohol use and smoking was possible.

## Conclusion

A majority of partners decreased their alcohol consumption in transition to parenthood, which seems to be a crucial time for changing alcohol-drinking patterns. The partners with higher AUDIT-C scores reported more support for decreased drinking. Most partners appreciated the midwife’s counseling about alcohol and pregnancy and those who filled out AUDIT in early pregnancy found the counseling to be more engaging. During pregnancy it is possible to detect partners with high alcohol consumption, and promote interventions for decreased drinking during pregnancy and parenthood, also for the partners.

## Abbreviations

ANC, antenatal care; AUDIT, Alcohol Use Disorders Identification Test: ten questions about alcohol consumption, risky drinking and dependency; AUDIT-C, a shorter form of the AUDIT questionnaire containing three questions about alcohol consumption; FASD, Fetal Alcohol Spectrum Disorder
